# Half-Dose versus Full Dose of Aprotinin in Cardiac Surgery: A Post-hoc Analysis of the Aprotinin European Registry

**DOI:** 10.1093/ejcts/ezaf260

**Published:** 2025-08-13

**Authors:** Pascal H Colson, Baptiste Gaudriot, Sophie Provenchère, Bertrand Rozec, Bernard Cholley, Philippe Mauriat, Marc Sénard, Jean-Luc Fellahi, NAPaR (Nordic Aprotinin Patient Registry) Investigators, Pascal H Colson, Baptiste Gaudriot, Sophie Provenchère, Bertrand Rozec, Bernard Cholley, Philippe Mauriat, Marc Sénard, Jean-Luc Fellahi

**Affiliations:** Département d’anesthésie-réanimation, CHU Montpellier, Avenue Doyen Giraud, Montpellier, 34295, France; CHU Rennes, 2 rue Henri-Le-Guilloux, Rennes, 35000, France; AP-HP, Bichat-Claude Bernard Hospital, 46 Rue Henri Huchard, Paris, 75018, France; CHU Nantes, Boulevard Jacques Monod, Nantes, 44000, France; Service d’Anesthésie-Réanimation, Hôpital Européen Georges Pompidou, AP-HP, 20 rue Leblanc, Paris, 75015, France; CHU Bordeaux, 1 Avenue de Magellsn, Bordeaux, 33600, France; CHU Liège, Avenue de l'Hôpital, Liège, 14000, Belgium; HCL, Louis Pradel Hospital, 59 Boulevard Pinel, Bron, 69677, France

**Keywords:** cardiac surgery, aprotinin, postoperative bleeding, registries

## Abstract

**Objectives:**

To compare the effectiveness and safety of the full and half doses of aprotinin, using the extended version of the Nordic Aprotinin Patient Registry, which was imposed by the European Medicines Agency following the reintroduction of aprotinin in cardiac surgery in the European market.

**Methods:**

A post-hoc analysis was performed on data prospectively collected from adult patients exposed to aprotinin during cardiac surgery, in 83 cardiovascular surgical centres in 9 European countries, from February 26, 2016 to October 5, 2022. Full-dose (FD) and half-dose (HD) regimens were used at the surgical team’s discretion. The FD loading, priming, and infusion doses were 2 MKIU, 2 MKIU, and 0.5 MKIU/h, respectively. Incidence of re-exploration for bleeding/tamponade 2 days after surgery (2 D-re-exploration), 7 D-mortality, 3 D-MACCE (major adverse cardiac and cerebral events), and 1 D-AKI (acute kidney injury) were compared between FD and HD patients using propensity score methods to reduce confounders.

**Results:**

A total of 2961 (44.4%) patients received the FD regimen and 3703 (55.6%) the HD regimen. After adjustment, the incidence of 2 D-re-exploration was estimated at 3.2% in the FD group and 4.4% in the HD group: OR [95% CI] = 0.70 [0.53-0.94] (*P* = .015). No difference in 7 D-mortality or 3 D-MACCE was observed between the 2 regimens. The incidence of 1 D-AKI was higher in patients with pre-operative renal dysfunction who received the FD regimen.

**Conclusions:**

Patients receiving the FD aprotinin regimen had less surgical re-exploration than those receiving the HD regimen. An increased risk of early postoperative AKI was observed in the FD group, mainly for patients with pre-operative chronic kidney disease.

## Graphical abstract

**Figure:**
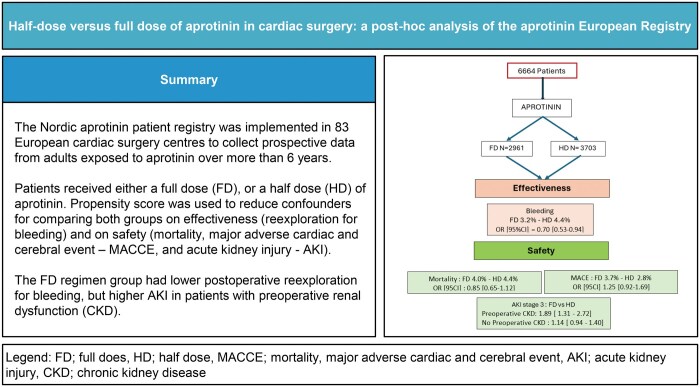

## INTRODUCTION

Antifibrinolytic treatment is recommended in the patient blood management approach for cardiac surgery.^1^^,^^2^ Aprotinin, which is indicated for prophylactic use to reduce blood loss and blood product transfusion in adults at high risk of major blood loss undergoing isolated coronary artery bypass graft surgery (iCABG), was reintroduced to the European market in February 2016 after the European Medicines Agency (EMA) lifted its marketing authorization suspension. The EMA required the new Marketing Authorization Holder to establish a registry to gather information on the pattern of aprotinin use and its safety outcomes.^3^

The Nordic Aprotinin Patient Registry (NAPaR) was implemented in several European cardiac surgery centres. It collected data from all adults exposed to aprotinin from February 26, 2016 to August 31, 2020. The Post-Authorization Safety Study (PASS), published in 2022, showed no safety signals.^3^ In France, the Health Authorities requested additional data on patients’ characteristics, as well as the effectiveness and safety of aprotinin. Therefore, data collection continued until October 5, 2022.^4^ Patients collected during this additional period in France were added to the NAPaR, resulting in an extended version (eNAPaR).

Two dosing regimens of aprotinin (full dose and half dose, FD and HD, respectively) were used at the discretion of the cardiac surgery centres. Before aprotinin’s suspension in 2008, several studies had explored dosing issues, but with relatively small populations.^5–7^ These studies generally favoured FD for reducing postoperative bleeding and transfusion.^6^^,^^7^ HD was inconsistently less effective than FD and was associated with potential cost savings.^5^^,^^8^^,^^9^ Additionally, the safety profiles of FD and HD regimens, particularly in high-risk patients, yielded conflicting results.^10–12^

The present analysis aimed to compare FD and HD aprotinin using the large cohort of eNAPaR data. The primary objective was to compare effectiveness, as assessed by the incidence of re-exploration for bleeding or tamponade. Secondary objectives evaluated the safety profile of aprotinin in terms of mortality, major adverse cardiac and cerebral events, and acute renal injury.

## METHODS

### Study design

The study is a post-hoc analysis of data collected in the eNAPaR from February 26, 2016 to October 5, 2022.

### Ethical considerations

As no ethical issues were raised by establishing a non-interventional registry, the study was not systematically submitted to relevant Independent Ethics Committees/Institutional Review Boards (IEC/IRB).^3^ However, the study protocol was submitted for approval to relevant IECs/IRBs in most countries and approvals obtained at national or local level depending on the country. According to local rules, before patients were included in the NAPaR, they could receive pertinent information about aprotinin (oral or written) and refuse data collection (oral).

### Patients

Adult patients were included in the eNAPaR if they were exposed to aprotinin during any on-pump cardiac surgery performed at one of the 83 participating centres from 9 European countries.

### Study procedures

Patients were treated according to the routine clinical practice of the centres. Aprotinin (Trasylol, Nordic Group B.V.) was used at the discretion of the investigator.

Aprotinin administration was a 4-step process: (1) administration of an initial test dose (0.01 million Kallikrein Inhibitor Units, MKIU) at least 10 min before the administration of the therapeutic dose; (2) slow administration of loading dose after induction of anaesthesia before sternotomy; (3) priming of the pump of the heart-lung machine by a solution containing aprotinin; and (4) constant infusion dose for the duration of the surgery. Except for the test dose designed to assess for potential allergic reaction, all other doses varied with the dose regimen. In the FD regimen, the loading dose was 2 MKIU, the priming dose was 2 MKIU, and the infusion dose was 0.5 MKIU/h. All these doses were divided by 2 in patients receiving the HD.

### Outcomes

The primary outcome was surgical re-exploration for postoperative bleeding (or tamponade) within 2 days of surgery (2 D-re-exploration). Secondary outcomes were (a) all-cause mortality on Day 7 (7 D-mortality); (b) major adverse cardiac and cerebral events (myocardial infarction or permanent stroke) occurring within 72 hours following surgery (3 D-MACCE); and (c) acute renal injury was assessed on KDIGO score based on changes in serum creatinine level within 1 day following surgery (1 D acute kidney injury [AKI]).

### Data collection

Data collected during routine clinical practice were entered into an electronic web-based record form.^3^ The registry prospectively captured information on the characteristics of the patients; the surgical procedure; the reasons and conditions of use of aprotinin; and safety outcomes occurring during cardiac surgery, the next 24 H, and during the hospital stay.

### Statistical analysis

A multivariate logistic regression model predicting the likelihood of receiving 1 of the 2 doses of aprotinin was used to calculate the propensity score (PS). Then, an inverse probability of treatment weighting (IPTW) method was used to adjust population (IPTW population).^13^^,^^14^ The weights used in the IPTW were determined using the PS: (1/PS) for HD and (1/[1-PS]) for FD. The PS was also used for matching patients (1:1) to generate a PS matched (PSM) population. For IPTW and PSM populations, standardized mean differences (SMD) between the 2 groups (FD and HD) were calculated and SMD < 0.1 assessed balance after IPTW.

The model was adjusted for potential confounders consisting in presurgical factors: sex (male/female); age (<75 years/≥75 years); body mass index (BMI, <25/≥25 kg.m^−2^); redo surgery (yes/no); renal dysfunction (creatinine clearance at baseline <50 ml/min or in dialysis; yes/no); active endocarditis (yes/no); active (not stopped before surgery) dual antiplatelet therapy (ADAT, aspirin, clopidogrel, prasugrel, ticagrelor, or cangrelor; yes/no); active anticoagulant (warfarin or phenprocoumon, unfractionated heparin, direct thrombin inhibitors, low molecular weight heparin, direct factor Xa inhibitors, any other medicine such as rivaroxaban, apixaban, or other direct factor Xa inhibitor; yes/no), emergency (elective/non-elective); iCABG (yes/no). “Country group” was not included in the model as in contingency tables performed during the modelling process, Cramer’s V coefficients were mainly above 0.5, indicating a strong association between this variable and some other variables, including the “dose regimen.” The country effect was subsequently assessed by a sensitivity analysis. Pre-operative variables with missing data rates above 20% were excluded.

Once the PSs were computed, the dose regimen, as well as all pre-operative (already included in the model) and per-operative factors (not included in the model), were analysed via univariate logistic regression analysis. This analysis which used IPTW or PSM data was aimed at evaluating if 1 of the 2 regimens (FD or HD) was associated with 2 D-re-exploration (primary outcome). Logistic regression was also used to identify pre- and per-operative factors associated with secondary outcomes and for the sensitivity analysis assessing the impact of the country groups on the outcomes. The Wald Chi-square test was used to compare FD and HD populations. The impact of pre-operative renal dysfunction on the relationship between dose regimens and 1 D-AKI was also assessed.

Missing values were not imputed, except for MACCE occurrence dates, which were assumed to be within 72 hours post-surgery when missing. This conservative approach ensured no events were missed but risked inaccurately including some events unrelated to aprotinin. A 2-sided *P*-value of <.05 was considered statistically significant. The robustness of statistically significant results was assessed using (i) a posteriori calculation of the fragility index (FI) and Fragility Quotient (FQ) as FI/sample size (FI was defined as the minimum number of patients whose status would need to change from event to non-event)^15^ and (ii) E-values (E) calculation to quantify the strength of unmeasured confounding necessary to negate the observed results.^16^ The analysis was performed using SAS (SAS Institute, Cary, North Carolina, USA), version 9.4.

## RESULTS

### Patients

**Figure 1.**
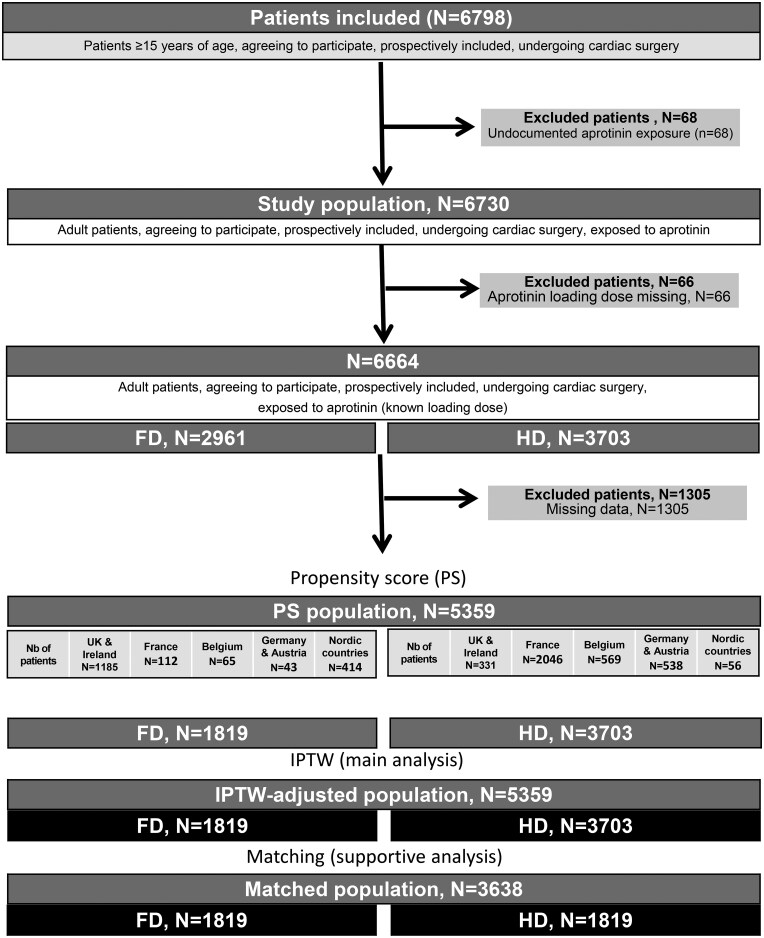
Flowchart. FD, full dose; HD, half dose; IPTW, inverse probability of treatment weighting

Data from 6798 patients were prospectively collected in the eNAPaR. Among them, 6664 were included in the *study population*, 2961 (44.4%) patients received FD, and 3703 (55.6%) received HD (**Figure 1**). Overall, 5359 patients (79.6% of the study population) had complete data allowing the construction of the PS (FD, *N* = 1819; HD, *N* = 3540). All these patients constituted the *analysed population* and *IPTW population* (details on centre and country distribution in **Tables S1 and S2**). The *PSM population* included 3638 patients (54.1% of the study population) (**Figure 1**; **Table S3**).

**Figure 2.**
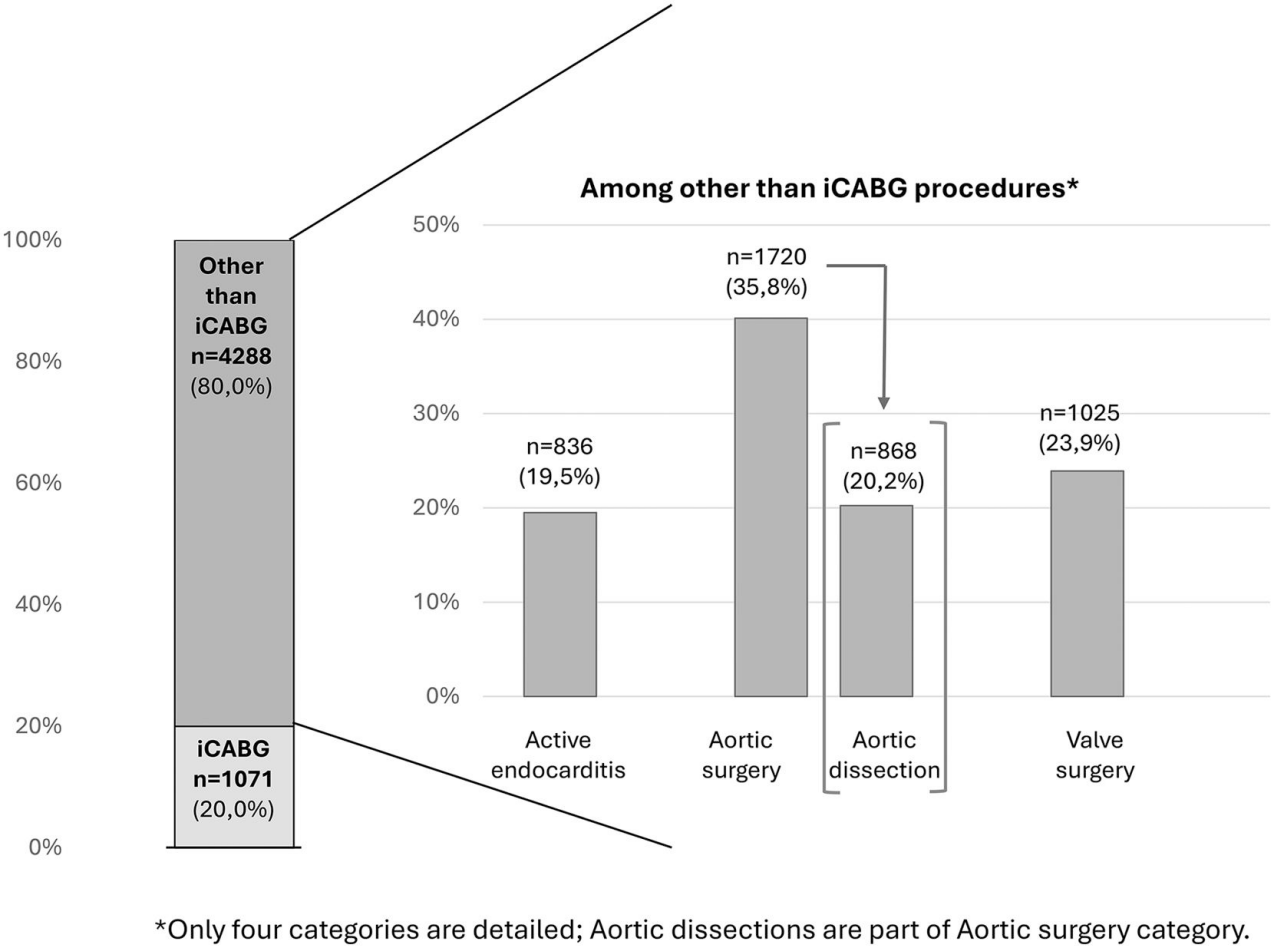
Characteristics of Non-isolated Coronary Artery Bypass Surgery Procedures (IPTW-Adjusted Population). Only 4 categories are detailed. Aortic dissections are part of aortic surgery category. iCABG, isolated coronary artery bypass graft surgery

In the *study population*, HD patients differed from FD patients. The IPTW balanced the 2 groups for variables included in the PS, and no significant difference was observed between FD and HD patients (**Table 1**). Similar results were observed in the *PSM population* (**Table S3**), with small differences (≤ 3%) between *IPTW* and *PSM populations* except for iCABG (8%). Incidences of non-iCABG surgery in study population are represented in **Figure 2**.

**Table 1.** Main Characteristics of Patients Before and After IPTW Adjustment

Patients’characteristics	Study population				IPTW-adjusted population				
	All	FD	HD	*P*-value	All	FD	HD	*P*-value	*SMD*
	N = 6730	N = 2961	N = 3703		N = 5359	N = 2674	N = 2685		
Female	1906 (28)	865 (29)	1020 (28)	.13	1486 (28)	737 (28)	749 (28)	.79	0.007
Elderly > 75 years	1175 (17)	502 (17)	660 (18)	.35	889 (17)	443 (17)	446 (17)	.95	0.002
BMI > 25 kg.m^−2^	4245 (63)	1935 (66)	2268 (61)	<.01	3311 (62)	1651 (62)	1660 (62)	.94	0.002
Renal dysfunction	1068 (16)	435 (15)	631 (17)	.01	868 (16)	438 (16)	431 (16)	.74	0.009
Active endocarditis	886 (14)	346 (13)	539 (15)	.01	839 (16)	419 (16)	419 (16)	.94	0.002
Active DAPT	579 (10)	195 (10)	383 (10)	.29	575 (11)	296 (11)	279 (10)	.40	0.023
Active anticoagulant	1885 (33)	621 (30)	1263 (34)	<.01	1800 (34)	914 (34)	886 (33)	.36	0.025
Redo surgery	2309 (35)	928 (31)	1367 (37)	<.01	2067 (39)	1032 (39)	1035 (39)	.97	0.0009
Non-elective surgery	3492 (52)	1569 (53)	1910 (52)	.25	2922 (55)	1489 (56)	1434 (53)	.09	0.046
iCABG	1549 (23)	643 (22)	881 (24)	.04	1071 (20)	529 (20)	542 (20)	.72	0.01

Number (%). BMI, body mass index; DAPT, dual antiplatelet therapy; FD, full dose; HD, half dose; iCABG, isolated coronary artery bypass graft, IPTW, inverse probability of treatment weighting; PS, propensity score; SMD, standardized mean difference.

### 2D-re-exploration for bleeding

**Figure 3.**
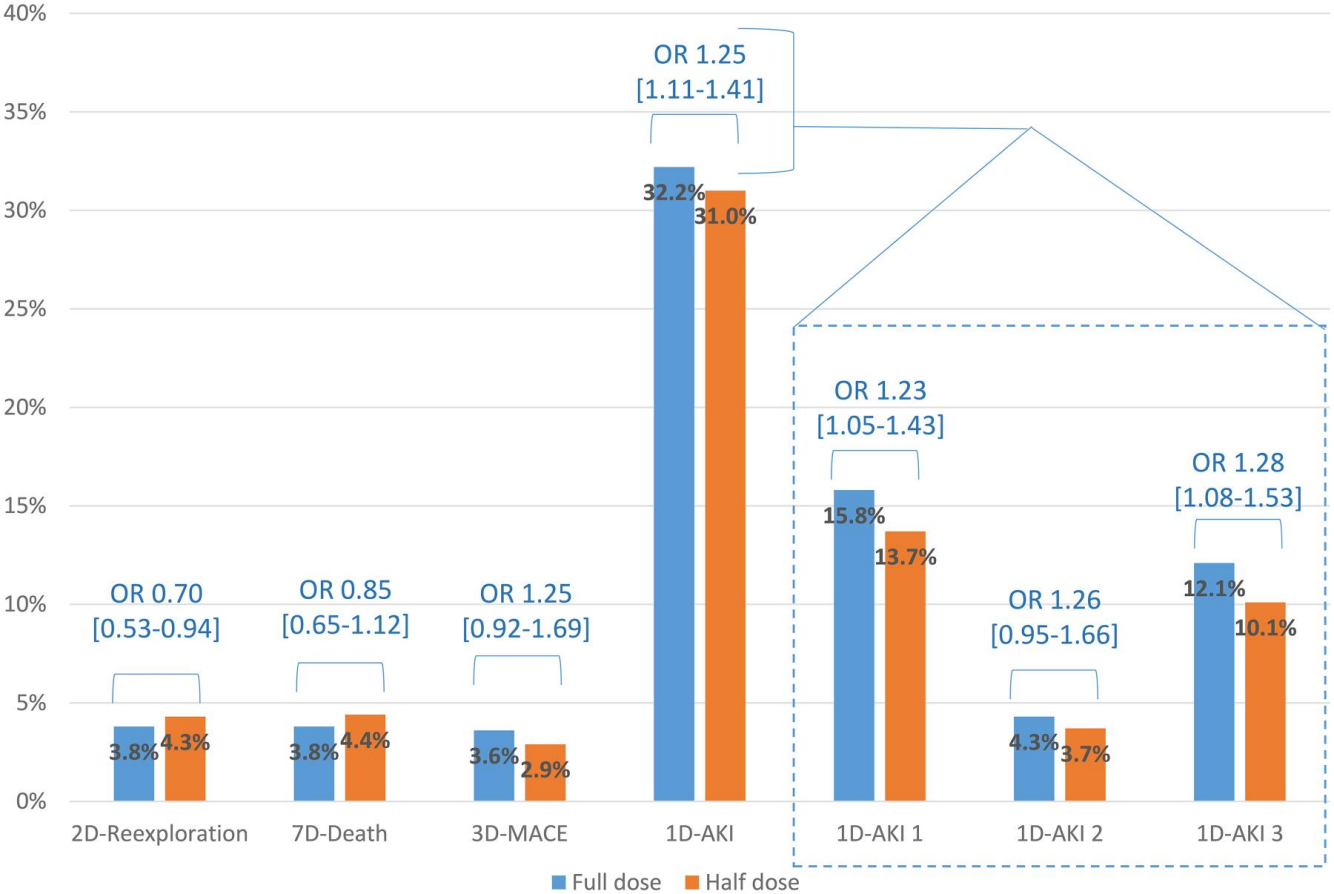
Bleeding and Safety Outcomes (IPTW-Adjusted Population). Bleeding was expressed as re-exploration for bleeding. MACCE, major adverse cardiac and cerebrovascular event; AKI, acute kidney injury according to KDIGO stages 1, 2, and 3; OR [95% CI], odds ratio [95% confidence interval]; 1 D, 2 D, 3 D, or 7 D, within 1, 2, 3, or 7 days

**Figure 4.**
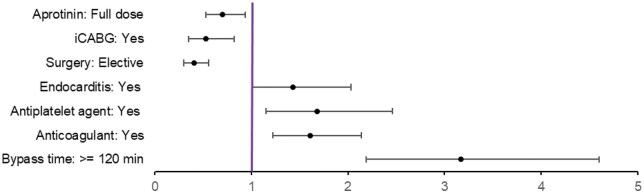
Factors Associated with 2 D-Re-exploration (IPTW-Adjusted Population). OR [95% CI], odds ratio [95% confidence interval]; iCABG, isolated coronary artery bypass graft surgery

In the *analysed population*, 203 (4.0%) patients were reexplored: 60 (3.3%) FD patients versus 152 (4.3%) HD patients. In the *IPTW population*, the percentage of patients who underwent 2 D-re-exploration was estimated at 3.2% for FD and 4.4% for HD: OR [95% CI] = 0.70 [0.53-0.94] (*P* = .015; E = 2.21) (**Figure 3**). The FI for this finding was 7 (FQ 0.13%). The risk of 2 D-re-exploration was lower in patients undergoing iCABG, or receiving FD aprotinin but increased with bypass time ≥120 min, emergency surgery, dual antiplatelet therapy, anticoagulant therapy, and endocarditis (**Figure 4**; **Table S4**). Similar findings were observed in the *PSM population* (**Table S5**). The sensitivity analysis showed no statistical association between aprotinin regimens and 2 D-re-exploration at the country level in the *IPTW population* (**Figure S1**). The absolute risk reduction with FD vs HD was 1.2%, meaning that re-exploration could be avoided with FD for 1 patient every 83 patients.

### Safety outcomes

7 D-mortality occurred in 219 (4.1%) patients: 71 (4.0%) FD patients versus 148 (4.2%) HD patients. In the *IPTW population*, the 7 D-mortality was 3.8% for FD and 4.4% for HD (*P* = .245) (**Figure 3**; **Figure S2**). In the *PSM population*, no significant association was observed between the dose regimen and the 7 D-mortality (**Table S6**).

A 3 D-MACCE was reported in 166 (3.1%) patients: 67 (3.7%) FD patients versus 99 (2.8%) HD patients. In the *IPTW population*, the incidence of 3 D-MACCE was estimated at 3.6% in the FD group and 2.9% in the HD group (*P* = .158) (**Figure 3**; **Figure S2**). In the *PSM population*, no significant association was found between the dose regimen and the occurrence of 3 D-MACCE (**Table S6**; **Figure S2**).

AKI KDIGO1, KDIGO2, and KDIGO3 occurred in 758 (14.2%), 206 (3.9%), and 571 (10.7%) patients, respectively. The proportions were 16.3%, 4.8%, and 13.1% for FD and 13.2%, 3.4%, and 9.5% for HD, respectively. In the *IPTW population*, the percentages of patients with any KDIGO, KDIGO1, KDIGO2, and KDIGO3 stage were 32.2%, 15.8%, 4.3%, and 12.1% for FD, and 27.5%, 13.7%, 3.7%, and 10.1% for HD, respectively (*P* < .01; E = 1.25; 1.76; 1.83; and 1.88) (**Figure 3**; **Figure S2**). The FI were 55 for any KDIGO (FQ 1.1%), 4 for KDIGO1 (FQ 0.08%), and 9 for KDIGO3 (FQ 0.17%). The association between the dose regimen and 1 D-AKI in patients increased with pre-operative renal dysfunction (**Table S7**). In the *PSM population*, the percentage of patients in each KDIGO class did not differ with the dose regimen (**Table S6**).

The sensitivity analysis showed no association between the aprotinin regimen and 7 D-mortality or 3 D-MACCE or AKI at the country level in the *IPTW population* except for France where an association was observed between dose regimen and 1 D-AKI (**Table S8**). **Figure S3** shows the logistic regression analysis of AKI factors in the *IPTW population.*

## DISCUSSION

The post-hoc analysis of the eNAPaR data showed that FD aprotinin was associated with fewer 2-day reexplorations compared to HD aprotinin. There were no differences between FD and HD patients in 7-day mortality and 3-day MACCE, but there was a higher incidence of 1-day AKI in FD patients.

The study population was derived from the largest database on aprotinin use in cardiac surgery since its reintroduction to the market in 2016. The benefit of FD over HD in terms of bleeding has been and remains a topic of debate.^5^^,^^8^^,^^9^ Since no randomized controlled trial (RCT) has been conducted yet, observational “real-life” data may offer an alternative analysis strategy to provide insights to guide dosing decisions.^17^ To mirror an RCT as closely as possible^18^ and overcome the inherent limitations of data extracted from the registry, statistical analyses were conducted with 2 methods of population adjustment. The IPTW population retained the entire cohort of the analysed population. The PSM population allowed for comparisons between smaller groups of patients, potentially yielding less consistent results than the IPTW population. However, PSM is more robust to the misspecification of the PS than the IPTW method.^13^^,^^14^ Both IPTW and PSM led to well-balanced covariates, thereby strengthening the validity of further causal differences observed between the 2 doses.

The incidence of postoperative re-exploration for bleeding is easily measured and frequently used as a quality indicator.^19^^,^^20^ The 2-D re-exploration rate observed in this study (4%) was close to the range reported in the literature (1%-3.1%),^21^^,^^22^ though the eNAPaR patients were at higher risk. These results are consistent with a meta-analysis showing that only FD aprotinin reduced the rate of re-exploration compared to placebo, HD aprotinin, or other antifibrinolytic drugs.^23^

Regarding safety, no differences were observed between aprotinin regimens for 7-day mortality and 3-day MACCE. These results align with data reported in the literature for aprotinin, tranexamic acid, or other fibrinolytic agents although the unusual, limited time windows may have missed some events.^10^^,^^12^^,^^22–24^ Other thrombotic events (eg, superficial and deep venous thrombosis, pulmonary embolism, acute limb ischaemia, and acute mesenteric ischaemia) have been reported more frequently with HD aprotinin than with tranexamic acid in a retrospective cohort study of a subset of patients at very high risk of bleeding, with high re-exploration rates (>15%).^12^ No significant difference in the occurrence of these events was reported in a previous large RCT comparing FD aprotinin with other antifibrinolytic drugs.^2^ Concerns regarding the risk of renal impairment with aprotinin use were raised in the early 2000s.^25–27^ In this study, AKI was defined according to the KDIGO classification, which is commonly used across surgical specialties.^28^ The eNAPaR registry allowed for serum creatinine collection up to 24 hours post-surgery, so AKI was assessed using the KDIGO classification at postoperative Day 1 only.^3^ AKI KDIGO stages 2 and 3 have been reported to occur within the first 24 postoperative hours.^29^ Therefore, the Day 1 AKI definition may have missed only a few postoperative AKI cases. In the IPTW population, FD was associated with an increased incidence of AKI, driven by KDIGO stage 1, representing almost half of the AKI incidence, and pre-operative renal dysfunction, a recognized factor of postoperative AKI.^28^^,^^30^

The study has several limitations. First, it relied on data collected from a registry and the possibility of treatment selection bias could not be fully excluded. Only collected variables could be analysed (eg, re-exploration for bleeding was reported, but bleeding volume was not), and some non-key pre-operative variables were excluded from the calculation of the PS due to missing data. The possibility that confounding factors were not considered in the analysis cannot be ruled out though e-values were calculated. FI were rather high compared to RCTs reported in high-impact medical journals; however, some FQ were low meaning small changes in the data could shift statistical significance. Second, most patients were from the United Kingdom or France, leading to overrepresentation of British and French practices and residual bias cannot be ruled out despite the absence of association between aprotinin regimens and 2 D-re-exploration at the country level depicted in forest plots. Finally, the observation period was close to the aprotinin administration (during surgery). This restrictive period may have missed later events.

This study also has strengths. It is based on a large, European, prospectively collected database of adults undergoing high-risk surgery, allowing for the comparison of the effectiveness and safety of FD and HD in real-world conditions. Additionally, adjustment of the study population by PS increased the study’s reliability.^15^^,^^31^

## CONCLUSION

In this post-hoc analysis of data prospectively collected in the eNAPaR, patients receiving FD aprotinin had fewer surgical reexplorations for bleeding at 2 days compared to those receiving HD aprotinin. There were no differences between FD and HD patients in 7-day mortality and 3-day MACCE, but there was a higher incidence of 1-day AKI in FD patients, mainly in case of pre-operative chronic kidney disease. A large multicentre randomized trial comparing tranexamic acid with aprotinin doses would be helpful to consolidate these results.

## Supplementary Material

ezaf260_Supplementary_Data

## Data Availability

The data and findings of this study are available upon reasonable request by contacting Nordic Pharma France (ma.france@nordicpharma.com).
