# Supplementary material for: Half-Dose versus Full Dose of Aprotinin in Cardiac Surgery: A Post-hoc Analysis of the Aprotinin European Registry
Source: Eur J Cardiothorac Surg. 2025 Aug 13;67(9):ezaf260. doi: 10.1093/ejcts/ezaf260 (PMC12410929; doi:10.1093/ejcts/ezaf260)
Supplement: ezaf260_Supplementary_Data [file ezaf260_supplementary_data.docx]

Supplementary data

**Table S1**

**Number of patients per site (N=5359 patients included in the IPTW method**

| **Country** | **Centre** | **Nb of patients** | **%** |
| --- | --- | --- | --- |
| **Austria** | **Kepler Universitätsklinikum GmbH, Linz** | **6** | **0.11** |
| **Austria** | **Krankenhaus Hietzing** | **4** | **0.08** |
| **Belgium** | **ASZ Aalst** | **9** | **0.17** |
| **Belgium** | **Imelda Ziekenhuis, Bonheiden** | **34** | **0.64** |
| **Belgium** | **OLV Aalst** | **24** | **0.45** |
| **Belgium** | **UZ Brussel Jette** | **1** | **0.02** |
| **Belgium** | **UZ Gasthuisberg Leuven** | **507** | **9.51** |
| **Belgium** | **UZ Gent** | **59** | **1.11** |
| **Germany** | **Herz-und Gefäß-Klinik GmbH Bad Neustadt/Saale** | **4** | **0.08** |
| **Germany** | **Herz-und Gefäßzentrum Bad Bevensen** | **81** | **1.52** |
| **Germany** | **Herzzentrum Dresden GmbH** | **428** | **8.03** |
| **Germany** | **Universitätsklinikum Giessen und Marburg, Standort Giessen, Klinik für Herz und Gefäßchirurgie** | **20** | **0.38** |
| **Germany** | **Universitätsklinikum Frankfurt** | **29** | **0.54** |
| **Germany** | **Universitätsklinikum des Saarlandes. Klinik für Thorax- und Herz-Gefäß-Chirurgie, Homburg** | **6** | **0.11** |
| **Germany** | **Westpfalzklinikum Kaiserslautern** | **3** | **0.06** |
| **Finland** | **Helsinki University Hospital** | **122** | **2.29** |
| **Finland** | **Kuopio University Hospital** | **13** | **0.24** |
| **Finland** | **Oulo University Hospital** | **49** | **0.92** |
| **Finland** | **Tampere University Hospital** | **4** | **0.08** |
| **Finland** | **Turku University Hospital** | **17** | **0.32** |
| **France** | **CH Annecy-Gennevois** | **65** | **1.22** |
| **France** | **CHRU Strasbourg** | **13** | **0.24** |
| **France** | **CHU Arnaud de Villeneuve, Montpellier** | **260** | **4.88** |
| **France** | **CHU Bordeaux** | **311** | **5.83** |
| **France** | **CHU Charles Nicolle, Rouen** | **101** | **1.89** |
| **France** | **CHU Dupuytren, Limoges** | **14** | **0.26** |
| **France** | **CHU Grenoble** | **44** | **0.83** |
| **France** | **CHU Nantes** | **156** | **2.93** |
| **France** | **CHU Rennes** | **151** | **2.83** |
| **France** | **CHU Dijon** | **109** | **2.05** |
| **France** | **CHU la Timone, Marseille** | **162** | **3.04** |
| **France** | **Clinique Saint-Pierre, Perpignan** | **43** | **0.81** |
| **France** | **Clinique de la Sauvegarde, Lyon** | **43** | **0.81** |
| **France** | **Clinique du Millnaire, Montpellier** | **10** | **0.19** |
| **France** | **Hopital Bichat, Paris** | **402** | **7.54** |
| **France** | **Hopital Europeen Georges Pompidou** | **26** | **0.49** |
| **France** | **Hopital Louis Pradel, Bron** | **64** | **1.20** |
| **France** | **Hopital Rangueil, Toulouse** | **46** | **0.86** |
| **France** | **Institut Arnault Tzanck, St laurent du var** | **45** | **0.84** |
| **France** | **La Pitie - Salpetriere, Paris** | **59** | **1.11** |
| **France** | **Robert Debre, CHU Reims** | **5** | **0.09** |
| **United Kingdom** | **Barts Heart Centre, London** | **48** | **0.90** |
| **United Kingdom** | **Basildon & Thurrock University Hospital** | **22** | **0.41** |
| **United Kingdom** | **Birmingham Childrens Hospital** | **1** | **0.02** |
| **United Kingdom** | **Bristol Royal infirmary** | **132** | **2.48** |
| **United Kingdom** | **Castle Hill Hospital, Hull** | **32** | **0.60** |
| **United Kingdom** | **Golden Jubilee Hospital, Glasgow** | **55** | **1.03** |
| **United Kingdom** | **Hammersmith Hospital, London** | **24** | **0.45** |
| **United Kingdom** | **Harefield Hospital** | **11** | **0.21** |
| **United Kingdom** | **John Radcliffe Hospital** | **27** | **0.51** |
| **United Kingdom** | **King's College hospital, London** | **85** | **1.59** |
| **United Kingdom** | **Leeds General Hospital** | **21** | **0.39** |
| **United Kingdom** | **Liverpool Heart & Chest Hospital** | **17** | **0.32** |
| **United Kingdom** | **London Bridge Hospital** | **2** | **0.04** |
| **United Kingdom** | **Manchester Heart Centre, Manchester Royal Infirmary** | **6** | **0.11** |
| **United Kingdom** | **Morriston Hospital, Swansea** | **2** | **0.04** |
| **United Kingdom** | **New Cross Hospital, Wolverhampton** | **254** | **4.77** |
| **United Kingdom** | **Northern General Hospital, Sheffield** | **17** | **0.32** |
| **United Kingdom** | **Nottingham City Hospital** | **99** | **1.86** |
| **United Kingdom** | **Papworth Hospital** | **145** | **2.72** |
| **United Kingdom** | **Queen Elizabeth Hospital, Birmingham** | **42** | **0.79** |
| **United Kingdom** | **Royal Brompton Hospital, London** | **203** | **3.81** |
| **United Kingdom** | **Royal Infirmary of Edinburgh** | **2** | **0.04** |
| **United Kingdom** | **Royal Stoke Hospital** | **49** | **0.92** |
| **United Kingdom** | **Royal Sussex Hospital, Brighton** | **1** | **0.02** |
| **United Kingdom** | **Southampton General Hospital** | **36** | **0.68** |
| **United Kingdom** | **St Thomas's Hospital, London** | **55** | **1.03** |
| **United Kingdom** | **University Hospital, Coventry** | **1** | **0.02** |
| **United Kingdom** | **University of South Manchester Hospital, Wythenshawe** | **66** | **1.24** |
| **United Kingdom** | **Victoria Hospital, Blackpool** | **58** | **1.09** |
| **Ireland** | **Mater Private, Dublin** | **1** | **0.02** |
| **Ireland** | **St James Hospital, Dublin** | **2** | **0.04** |
| **Norway** | **Trondheim University Hospital, Trondheim** | **3** | **0.06** |
| **Sweden** | **Karolinska Universitetsjukhuset Solna (Stockholm)** | **200** | **3.75** |
| **Sweden** | **SkÃ¥nes Universitetsjukhus Lund** | **1** | **0.02** |
| **Sweden** | **UmeÃ¥ University Hospital** | **41** | **0.77** |
| **Sweden** | **Universitetsjukhuset Orebro** | **20** | **0.38** |

**Table S2**

**Indication and dose regimens by country (analysed population)**

| **Countries** | **Centres** | **Patients** | **Aprotinin dose regimen** | | **Cardiac surgery procedure** | |
| --- | --- | --- | --- | --- | --- | --- |
|  | **(N)** | **N=5359** | **FD** | **HD** | **iCABG** | **Non-iCABG** |
| Ireland | 2 | 3 (0.1) | 3 (100) | 0 (0) | 0 (0) | 3 (100) |
| United Kingdom | 29 | 1513 (28.2) | 1182 (78.1) | 331 (21.9) | 118 (7.8) | 1395 (92.2) |
| United Kingdom - Ireland | 31 | 1516 (28.3) | 1185 (78.2) | 331 (21.8) | 118 (7.8) | 1398 (92.2) |
| France | 21 | 2158 (40.3) | 112 (5.2) | 2046 (94.8) | 314 (14.5) | 1844 (85.4) |
| Belgium | 6 | 634 (11.8) | 65 (10.3) | 569 (89.7) | 17 (2.68) | 617 (97.3) |
| Austria | 2 | 10 (0.2) | 2 (20.0) | 8 (80.0) | 8 (80.0) | 2 (20.0) |
| Germany | 7 | 571 (10.6) | 41 (7.2 | 530 (92.8) | 570 (99.8) | 1 (0.2) |
| Germany - Austria | 9 | 581 (10.8) | 43 (7.4) | 538 (92.6) | 578 (99.5) | 3 (0.5) |
| Finland | 5 | 205 (3.8) | 151 (73.7) | 54 (26.3) | 30 (14.6) | 175 (85.4) |
| Norway | 1 | 3 (0.1) | 3 (100) | 0 (0) | 0 (0) | 3 (100) |
| Sweden | 4 | 262 (4.9) | 260 (99.2) | 2 (0.8) | 27 (10.3) | 235 (89.7) |
| Nordic countries | 10 | 470 (8.8) | 414 (88.1) | 56 (11.9) | 57 (12.1) | 413 (87.9) |

Data expressed as numbers (percent); FD: full dose; HD: half dose; iCABG : isolated coronary artery bypass surgery.

Countries were classified into five groups based on similar profiles of patients regarding aprotinin dose (FD or HD) and indication (iCABG or other): (1) UK and Ireland; (2) France; (3) Belgium; (4) Germany and Austria; (5) Nordic countries (Finland, Norway, and Sweden).

**Table S3**

**Main characteristics of adult patients exposed to aprotinin during cardiac surgery before and after matching**

| **Patients characteristics** | **Before PS (Study population)** | | | | **After PS-matching (Matched population)** | | | | | |
| --- | --- | --- | --- | --- | --- | --- | --- | --- | --- | --- |
|  | **All** | **FD** | **HD** | ***P-value*** | | **All** | **FD** | **HD** | ***P-value*** | ***SMD*** |
|  | N=6730 | N=2961 | N=3703 |  |  | N=3638 | N=1819 | N=1819 |  |  |
| Female | 1906 (28) | 865  (29) | 1020 (28) | 0.13 | | 1060 (29) | 526  (29) | 534  (29) | 0.770 | 0.01 |
| Elderly >75 years | 1175 (17) | 502  (17) | 660  (18) | 0.35 | | 502  (14) | 256  (14) | 246  (14) | 0.631 | 0.02 |
| BMI >25 kg.m^-2^ | 4245 (63) | 1935 (66) | 2268 (61) | <0.01 | | 2273 (62) | 1139 (63) | 1134 (62) | 0.864 | <0.01 |
| Renal dysfunction | 1068 (16) | 435  (15) | 631  (17) | 0.01 | | 486  (13) | 258  (14) | 228  (13) | 0.144 | 0.05 |
| Active endocarditis | 886  (14) | 346  (13) | 539  (15) | 0.01 | | 611  (17) | 317  (17) | 294  (16) | 0.308 | 0.03 |
| Active DAPT | 579  (10) | 195  (10) | 383  (10) | 0.29 | | 353  (10) | 176  (10) | 177  (10) | 0.955 | <0.01 |
| Active anticoagulant | 1885 (33) | 621  (30) | 1263 (34%) | <0.01 | | 1117 (31) | 548  (30) | 569  (31) | 0.450 | 0.02 |
| Redo surgery | 2309 (35) | 928  (31) | 1367 (37) | <0.01 | | 1525 (42) | 762  (42) | 763  (42) | 0.973 | <0.01 |
| Non-elective surgery | 3492 (52) | 1569 (53) | 1910 (52) | 0.25 | | 2030 (56) | 1027 (56) | 1003 (55) | 0.423 | 0.03 |
| iCABG | 1549 (23) | 643  (22) | 881  (24) | 0.04 | | 436  (12) | 218  (12) | 218  (12) | 1.000 | 0 |

Data expressed as numbers (percent); BMI: body mass index; DAPT: dual antiplatelet therapy; FD: full dose; HD: half dose; iCABG: isolated coronary artery bypass graft, IPTW: inverse probability of treatment weighting; PS: propensity score; SMD: standardized mean difference.

**Table S4**

**Factors associated with reexploration (2D-reexploration) (IPTW-adjusted population)**

|  | | No N=4979 | Yes N=205 | ALL N=5184 | OR  [IC95%] | PValue |  |  |  |  |  |  |
| --- | --- | --- | --- | --- | --- | --- | --- | --- | --- | --- | --- | --- |
| Bypass time | <120 min | 2019.84 (98.30%) | 34.84(1.70%) | 2054.68 | Ref | . |  |  |  |  |  |  |
|  | >= 120 min | 2931.90 (94.81%) | 160.47 (5.19%) | 3092.37 | 3.40[ 2.32 - 4.98] | <.001 |  |  |  |  |  |  |
| Emergency | No | 2771.88 (94.85%) | 150.56 (5.15%) | 2922.45 | Ref | . |  |  |  |  |  |  |
|  | Yes | 2383.92 (97.84%) | 52.63 (2.16%) | 2436.55 | 0.41 [ 0.30 - 0.56] | <0.001 |  |  |  |  |  |  |
| DAPT | No | 4613.89 (96.45%) | 169.76 (3.55%) | 4783.64 | Ref | . |  |  |  |  |  |  |
|  | Yes | 541.92 (94.19%) | 33.44 (5.81%) | 575.36 | 1.68 [ 1.15 - 2.46] | 0.008 |  |  |  |  |  |  |
| Anticoagulant | No | 3446.57 (96.83%) | 112.83 (3.17%) | 3559.40 | Ref | . |  |  |  |  |  |  |
|  | Yes | 1709.24 (94.98%) | 90.36 (5.02%) | 1799.60 | 1.61 [ 1.22 - 2.14] | <0.001 |  |  |  |  |  |  |
| Aprotinin | Half dose | 2566.53 (95.58%) | 118.82 (4.42%) | 2685.35 | Ref | . |  |  |  |  |  |  |
|  | Full dose | 2589.28 (96.84%) | 84.37 (3.16%) | 2673.65 | 0.70 [ 0.53 - 0.94] | <0.015 |  |  |  |  |  |  |
| iCABG | No | 4109.38 (95.83%) | 178.92 (4.17%) | 4288.30 | Ref | . |  |  |  |  |  |  |
|  | Yes | 1046.42 (97.73%) | 24.28 (2.27%) | 1070.70 | 0.53 [0.35-0.82] | 0.004 |  |  |  |  |  |  |
| Endocarditis | No | 4359.42 (96.44%) | 161.06 (3.56%) | 4520.48 | Ref | . |  |  |  |  |  |  |
|  | Yes | 796.39 (94.98%) | 42.13 (5.02%) | 838.52 | 1.43 [1.01-2.03] | 0.043 |  |  |  |  |  | . |

Data expressed as numbers (percent); DAPT: dual antiplatelet therapy; iCABG: isolated coronary artery bypass graft, IPTW: inverse probability of treatment weighting.

**Table S5**

**Factors associated with reexploration for bleeding or tamponade within the first 48 hours (2D-reexploration) - (matched population)**

|  | | **No (N=3489)** | **Yes (N=149)** | **ALL (N=3638)** | **OR [95CI]** | **P-value** |
| --- | --- | --- | --- | --- | --- | --- |
| **CPB time** | <120 min | 1221 (98.5) | 19 (1.5) | 1240 | Ref. |  |
|  | ≥120 min | 2134 (94.5) | 124 (5.5) | 2258 | 3.73 [2.29-6.08] | <0.0001 |
| **Aprotinin Dose** | HD | 1730 (95.1) | 89 (4.9) | 1819 | Ref. |  |
|  | FD | 1759 (96.7) | 60 (3.3) | 1819 | 0.66 [0.47-0.93] | 0.016 |
| **Emergency** | Non-elective | 1920 (94.6) | 110 (5.4) | 2030 | Ref. |  |
|  | Elective | 1569 (97.6) | 39 (2.4) | 1608 | 0.43 [0.30-0.63] | <0.0001 |
| **Active anticoagulant** | No | 2438 (96.7) | 83 (3.3) | 2521 | Ref. |  |
|  | Yes | 1051 (94.1) | 66 (5.9) | 1117 | 1.84 [1.32-2.57] | <0.0001 |

Data expressed as numbers (percent); OR [95CI]: odds ratio [95% confidence interval]; CPB: cardiopulmonary bypass; FD: full dose; HD: half dose; min: minute; Ref.: reference.

**Table S6**

**Factors associated with safety outcomes (matched population)**

| **Aprotinin  dose** | | **No** | | | | | **Yes** | | | | | **All** | **OR [95CI]** | | | | **P-value** | | |  |  |  |  |  |
| --- | --- | --- | --- | --- | --- | --- | --- | --- | --- | --- | --- | --- | --- | --- | --- | --- | --- | --- | --- | --- | --- | --- | --- | --- |
| **7D-Death** | |  | | | | |  | | | | |  |  | | | |  | | |  |  |  |  |  |
| All | | 3366 (95.5) | | | | | 160 (4.5) | | | | | 3526 |  | | | | 0.146 | | |  |  |  |  |  |
| FD | | 1692 (96.0) | | | | | 71 (4.0) | | | | | 1763 | 0.79 [0.57-1.09] | | | |  | | |  |  |  |  |  |
| HD | | 1674 (94.9) | | | | | 89 (5.1) | | | | | 1763 | Ref. | | | |  | | |  |  |  |  |  |
| **3D-MACCE** | |  | | | | |  | | | | |  |  | | | |  | | |  |  |  |  |  |
| All | | 3502 (96.6) | | | | | 124 (3.4) | | | | | 3626 |  | | | | 0.361 | | |  |  |  |  |  |
| FD | | 1746 (96.3) | | | | | 67 (3.7) | | | | | 1813 | 1.18 [0.83-1.69] | | | |  | | |  |  |  |  |  |
| HD | | 1756 (96.9) | | | | | 57 (3.1) | | | | | 1813 | Ref. | | | |  | | |  |  |  |  |  |
| **1D-AKI** | | | **KDIGO 0** | | | **KDIGO 1,2,3** | | | |  | | | | |  | | |  | |  |  |  |  |  |
| All | | 2417 (67.4) | | | | | 1167 (32.6) | | | | | 3584 |  | | | | 0.04 | | |  |  |  |  |  |
| FD | | 1180 (65.9) | | | | | 612 (34.2) | | | | | 1792 | 1.16 [1.01-1.33] | | | |  | | |  |  |  |  |  |
| HD | | 1237 (69.0) | | | | | 555 (31.0) | | | | | 1792 | Ref. | | | |  | | |  |  |  |  |  |
| **1D-AKI** |  | | | **KDIGO** | | | | |  | |  | | |  | |  | | |  |  |  |  |  |  |
|  | **No** | | | **1** | **2** | | | **3** | **All** | | **1 vs 0**  **OR [95CI]** | | | **2 vs 0**  **OR [95CI]** | | **3 vs 0**  **OR [95CI]** | | | **P-value** |  |  |  |  |  |
| All | 2417 (67.4) | | | 563  (15.7) | 158  (4.4) | | | 446  (12.4) | 3584 | |  | | |  | |  | | | 0.217 |  |  |  |  |  |
| FD | 1180 (65.8) | | | 292  (16.3) | 86  (4.8) | | | 234  (13.1) | 1792 | | 1.13  [0.94-1.36] | | | 1.25  [0.91-1.73] | | 1.16  [0.95-1.42] | | |  |  |  |  |  |  |
| HD | 1237 (69.0) | | | 271  (15.1) | 72  (4.0) | | | 212  (11.8) | 1792 | | Ref. | | | Ref. | | Ref. | | |  |  |  |  |  |  |

Data expressed as numbers (percent); 1D, 3D, or 7D: within the first 24 hours, 72 hours, or 7 days; AKI: acute kidney injury (KDIGO stages 1 ,2, 3); D: day; FD: full dose; HD: half dose; IPTW: inverse probability of treatment weighting; KDIGO: kidney disease, improving global outcomes; MACCE: major adverse cardiac and cerebrovascular event; OR [95CI]: odds ratio [95% confidence interval]; Ref.: reference.

**Table S7**

**Impact of preoperative renal dysfunction (IPTW-adjusted population)**

| **Preoperative renal dysfunction§** | **Aprotinin dose** | **OR [95CI]** | | |  | **P-value** | |
| --- | --- | --- | --- | --- | --- | --- | --- |
|  |  | **KDIGO 0 vs KDIGO 1,2,3** | | | | |  |
| **Yes** | FD vs HD | 1.71  [ 1.29 - 2.27] | | | | | 0.001 |
| **No** | FD vs HD | 1.17  [ 1.03 - 1.33] | | | | | 0.017 |
|  |  | **AKI 1 vs 0** | **AKI 2 vs 0** | **AKI 3 vs 0** | | **P-value** | |
| **Yes** | FD vs HD | 1.47  [ 1.02 - 2.14] | 3.22  [ 0.83 - 12.45] | 1.89  [ 1.31 - 2.72] | | 0.001 | |
| **No** | FD vs HD | 1.19  [ 1.00 - 1.41] | 1.18  [ 0.89 - 1.57] | 1.14  [ 0.94 - 1.40] | | 0.122 | |

OR [95CI]: odds ratio [95% confidence interval]; AKI: acute kidney injury (KDIGO stages 1 ,2, 3); FD: full dose; HD: half dose; IPTW: inverse probability of treatment weighting
§= creatinine clearance < 50 ml/min or dialysis.

**Table S8**

**Factors associated with safety outcomes - By country (IPTW-adjusted population)**

| **Mortality and MACCE** | | **Aprotinin dose** | | | **OR [CI95%]** | | **P-value*** |
| --- | --- | --- | --- | --- | --- | --- | --- |
| **7D-mortality** | |  | | |  | |  |
| United Kingdom – Ireland | | FD vs HD | | | 0.64 [0.37 – 1.13] | | 0.13 |
| Belgium | | FD vs HD | | | 1.26 [0.21 – 7.41] | | 0.80 |
| France | | FD vs HD | | | 1.27 [0.70 – 2.29] | | 0.44 |
| Nordic countries | | FD vs HD | | | 1.55 [0.17 – 13.89] | | 0.69 |
| Germany – Austria | | FD vs HD | | |  | | NC |
| **3D-MACCE** | |  | | |  | |  |
| United Kingdom – Ireland | | FD vs HD | | | 0.67 [0.38 – 1.20] | | 0.18 |
| Belgium | | FD vs HD | | | 1.16 [0.18 – 7.61] | | 0.87 |
| France | | FD vs HD | | | 0.86 [0.34 – 2.18] | | 0.75 |
| Nordic countries | | FD vs HD | | | 1.25 [0.22 – 6.99] | | 0.80 |
| Germany – Austria | | FD vs HD | | | NC | | 0.96 |
| **1D-AKI** |  | | **1 vs 0** | **2 vs 0** | | **3 vs 0** | **P-value** |
| United Kingdom - Ireland | FD vs HD | | 0.82  [0.59 - 1.15] | 2.40  [0.96 - 5.97] | | 1.12  [0.74 - 1.70] | 0.13 |
| Belgium | FD vs HD | | 1.86  [0.93 - 3.71] | 2.80  [0.66 - 11.77] | | 2.05  [0.80 - 5.23] | 0.09 |
| France | FD vs HD | | 0.69  [0.43 - 1.11] | 1.33  [0.73 - 2.40] | | 0.52  [0.29 - 0.92] | 0.04 |
| Nordic countries | FD vs HD | | 1.46  [0.59 - 3.64] | 0.73  [0.19 - 2.84] | | 4.74  [0.97 - 23.14] | 0.21 |
| Germany - Austria | FD vs HD | | 1.42  [0.47 - 4.29] | NC | | 1.35  [0.26 - 7.10] | 0.92 |

OR [95CI]: odds ratio [95% confidence interval]; AKI: acute kidney injury (stage 0, 1,2, 3); D: day; FD: full dose; HD: half dose; IPTW: inverse probability of treatment weighting; MACCE: major adverse cardiac and cerebrovascular event; NC: not calculated;
Countries were classified into five groups based on similar profiles of patients regarding aprotinin dose (FD or HD) and indication (iCABG or other): (1) UK and Ireland; (2) France; (3) Belgium; (4) Germany and Austria; (5) Nordic countries (Finland, Norway, and Sweden)

**Figure 1S**

2D-reexploration for bleeding: Aprotinin Full Dose versus Half Dose - By country (IPTW-adjusted population);

OR [95CI]: odds ratio [95% confidence interval]**.**

Countries were classified into five groups based on similar profiles of patients regarding aprotinin dose (FD or HD) and indication (iCABG or other): (1) UK and Ireland; (2) France; (3) Belgium; (4) Germany and Austria; (5) Nordic countries (Finland, Norway, and Sweden)

**Figure 2S**

Safety outcomes: Aprotinin Full Dose versus Half Dose (IPTW-adjusted population)

OR [95CI]: odds ratio [95% confidence interval].

MACCE: major adverse cardiac and cerebrovascular event; AKI: acute kidney injury (stage 1,2,3)

Figure 3S

Acute Kidney Injury: risk factors (IPTW-adjusted population)

OR [95CI]: odds ratio [95% confidence interval]; iCABG: isolated coronary artery bypass graft surgery; BMI: body mass index; CKD: chronic kidney disease
